# Nanoliposomes containing three essential oils from the *Artemisia* genus as effective larvicides against *Aedes aegypti* and *Anopheles stephensi*

**DOI:** 10.1038/s41598-023-38284-6

**Published:** 2023-07-07

**Authors:** Alireza Sanei-Dehkordi, Abdolmajid Ghasemian, Elham Zarenezhad, Hajar Qasemi, Mahdi Nasiri, Mahmoud Osanloo

**Affiliations:** 1 Department of Medical Entomology and Vector Control, School of Health, Hormozgan University of Medical Sciences, Bandar Abbas, Iran; 2 Infectious and Tropical Diseases Research Center, Hormozgan Health Institute, Hormozgan University of Medical Sciences, Bandar Abbas, Iran; 3 Noncommunicable Diseases Research Center, Fasa University of Medical Sciences, Fasa, Iran; 4 Department of Medical Biotechnology, School of Advanced Technologies in Medicine, Fasa University of Medical Sciences, Fasa, Iran; 5 Department of Medical Nanotechnology, School of Advanced Technologies in Medicine, Fasa University of Medical Sciences, Fasa, Iran

**Keywords:** Nanoscience and technology, Disease prevention

## Abstract

*Aedes aegypti* and *Anopheles stephensi* have challenged human health by transmitting several infectious disease agents, such as malaria, dengue fever, and yellow fever. Larvicides, especially in endemic regions, is an effective approach to the control of mosquito-borne diseases. In this study, the composition of three essential oil from the *Artemisia* L. family was analyzed by Gas Chromatography–Mass Spectrometry. Afterward, nanoliposomes containing essential oils of *A. annua*, *A. dracunculus*, and *A. sieberi* with particle sizes of 137 ± 5, 151 ± 6, and 92 ± 5 nm were prepared. Besides, their zeta potential values were obtained at 32 ± 0.5, 32 ± 0.6, and 43 ± 1.7 mV. ATR-FTIR analysis (Attenuated Total Reflection-Fourier Transform InfraRed) confirmed the successful loading of the essential oils. Moreover, The LC_50_ values of nanoliposomes against *Ae. aegypti* larvae were 34, 151, and 197 µg/mL. These values for *An.stephensi* were obtained as 23 and 90, and 140 µg/mL, respectively. The results revealed that nanoliposomes containing *A. dracunculus* exerted the highest potential larvicidal effect against *Ae. aegypti* and *An. stephensi,* which can be considered against other mosquitoes.

## Introduction

Mosquitoes transmit several pathogens to humans, e.g., the *Aedes* genus can transmit Flaviviruses (mostly Dengue and Japanese encephalitis), Togaviruses, and Bunyaviruses^1,2^. These vectors are distributed worldwide, especially in tropical and temperate regions. *Aedes aegypti* is the most efficient vector of several arboviruses, such as dengue, yellow fever, Zika, and chikungunya^3,4^. It is an important threat to human health, e.g., 100 million people are affected annually by symptomatic Dengue virus infection^5^.

According to the WHO report, nearly 90 countries are contaminated by malaria, with more than 600,000 death in 2021^6,7^. *Anopheles stephensi* as the main vector of malaria needs to be controlled^8,9^. However, the vector has been endemic to Arabia Peninsula and Southeast Asia since its first emergence in Djibouti in 2012. Its geographical distribution has extended to African and Asian countries and five continents of the world^10,11^. Various inequalities such as climate change, globalization, international transport, population movements, international conflicts, and socioeconomic conditions alongside the vector resistance to WHO-recommended pesticides have affected the vector's residence and spread^12,13^.

Plant-based compounds (Extract and Essential oil (EO)) have been applied as pesticides (larvicidal and adulticidal) for many years^14^. These compounds are biodegradable, highly effective, non-toxic to the host, and target-specific^15,16^. Moreover, the lack of bioinsecticide resistance is another benefit of phytochemicals^17^. For instance, *Artemisia* L. is a widespread genus of the *Asteraceae* family; its 500 species are mainly found in Asia, Europe, and North America^18,19^. *Artemisia*-derived EOs contain high amounts of terpenes; they are thus frequently utilized as insecticides^20,21^. For instance, the EO of *A. annua* showed efficient insecticidal properties against red flour beetle (*Tribolium casteneum*)^22^. Likewise, the insecticidal effect of *A. dracunculus* L. EO on *Aphis gossypii* was reported^23^. Moreover, the toxic and repellent potential of *A. sieberi* against *Dermanyssus gallinae* (poultry red mite) was reported^24^.

Nanoliposome are bilayered vesicles containing amphiphilic molecules similar to cell membranes; stability, durability, potency, and efficacy of the entrapped compounds in nanoliposomes are improved^25–27^. Besides, some reports on applying nanoliposomes containing EOs as larvicides have been reported. For instance, nanoliposomes containing *Citrus aurantium* EO with LC_50_ value of 4.9 µg/ml against *Culex quinquefasciatus*^28^*.* Moreover, nanoliposomes containing carvacrol with an LC_50_ value of 128 µg/mL against *An. stephensi*^29^. However, to the authors’ best knowledge, no report was available on the use of nanoliposomes containing EO as larvicides against *Ae. aegypti*.

For the first time, nanoliposomes containing *A. annua*, *A. dracunculus*, and *A. sieberi* EOs were proposed in the current study. Moreover, their larvicidal properties against *Ae. aegypti* and *An. stephensi* was investigated.

## Materials and methods

### Materials

Tween 20, cholesterol, and egg lecithin were obtained from Merck Chemicals Co. (Germany). The Culicidae insectary at Hormozgan University Medical Sciences supplied *An. stephensi* and *Ae. aegypti* larvae; they were continually available for bioassay testing. Besides, the mosquito colonies were maintained at relative humidity (70 ± 5%), with photoperiod cycles of 12:12 (light:dark) at 27 ± 1 °C. Moreover, the polytetrafluoroethylene (PTFE)-based membrane method was used to blood-feed adult female mosquitoes^30^.

### Chemical compositions of the EOs

The chemical compound of the EOs was performed using an Agilent type 6890 GC–MS device equipped with a BPX5 silica capillary column (30 m × 0.25 μm, layer thickness of 0.25 μm) as described in our previous report^31^. To identify the EO's constituent compositions, 1 µL *n-*hexane was added in column chromatography. The temperature was scheduled; the oven temperature was set to 50 °C for 5 min. Then, the temperature was increased to 240 °C at a rate of 3 °C min^−1^, in continue, the temperature was increased to 300 °C at a rate of 15 °C min^−1^ for 3 min. Finally, the transfer line temperature was adjusted to 250 °C by split 1 to 35. Helium was used as the carrier gas at a flow rate of 0.5 mL min^−1^. The mass spectrometer (Agilent 5973 model) was scanned between 40 to 500 amu with an ionization voltage of 70 eV and ionization source temperature of 220 °C. The software used was Chemstation. Identification of the spectra was done with the help of their inhibition index and its comparison with the indices found in the source books and papers, using the mass spectra of standard compounds and the information available in the computer and virtual library^32^.

### Preparation of nanoliposomes containing EOs

Nanoliposomes were prepared using ethanol injection method^28^. The mixture of lecithin (3% w/v), cholesterol (0.5% w/v), tween 20 (2% w/v), and each EO (2% w/v) was added in absolute ethanol and stirred overnight (2000 RPM) to oily phase prepared. After that, 1 mL of the oily phase was added to the 4 mL of distilled water. Finally, the mixture was mixed for 1 h at 2000 RPM and room temperature to stabilize the formed nanoliposomes. Meanwhile, free nanoliposomes were prepared using the above method but without EO.

### Investigation of size and zeta potential of the nanoliposomes

Three prepared nanoliposomes and free nanoliposomes were poured into a quartz cell and transferred to the DLS machine to investigate particle size and particle size distribution (SPAN). The SPAN was calculated through the following equation: d90 − d10/d50. Also, the zeta potential of the samples was measured at room temperature^33^.

### Investigation loading of the EOs in nanoliposomes

The loading of EOs was confirmed by ATR-FTIR qualitative method. For this purpose, each EO, free liposome, and nanoliposome containing each EO was subjected to the FTIR machine, and spectra in the 400 to 4000 wavenumber cm^−1^ were recorded^34^.

### Mosquito rearing and larvicidal bioassays

The World Health Organization-recommended protocol was applied for the larvicidal bioassay tests^35^. In a 400 mL beaker containing 200 mL water, 25 larvae of *An. stephensi* or *Ae. aegypti* in the late third and early fourth instars were subjected to 12.5, 25, 50, 100, and 20 µg/mL of nanoliposome containing each EO. Besides, 1 mL of ethanol and free nanoliposomes were added to three bakers as control and negative control groups. Larval mortality was then noted after 24 h exposure. Three replicates for larvicidal bioassay were carried out, and larvicidal effects are presented as mean ± standard deviations. Besides, LC_50_ values with upper and lower confidence limits were calculated using the CalcuSyn software (free version). The non-overlap between the samples’ upper and lower confidence limits was considered a significant difference.

### Ethical approval

The ethics committee has approved this research at Fasa University of Medical Sciences, Iran, IR.FUMS.REC.1401.145. Besides, this study did not include human investigation, so consent to participate is not applicable.

## Results

### Identified compounds in the EOs

Identified compounds in the EOs are listed in Table 1. Artemisia ketone (26.2%), camphor (19.2%), 1,8-cineole (12.3%), trans-caryophyllene (4.5%), and camphene (4.4%) are five major compounds of *A. annua* EO. Besides, estragole (67.6%), *cis*-ocimene (8.7%), ɣ-terpinene (7.6%), *trans*-ocimene (4.3%), and α-pinene (1.6%) are five major compounds in *A. dracunculus* EO. Moreover, camphor (28.3%), β-thujone (15.9%), α-thujone (8.4%), 1,8-cineole (1.8%), and borneol (4.2%) are five major compounds in *A. sieberi* EO.

**Table 1:** Identified compounds using GC–MS analysis in the used EOs.

Compound	Retention Index	*A. annua*		*A. dracunculus*		*A. sieberi*	
		Area	%	Area	%	Area	%
α-pinene	932	122,296,938	4.1	513,847,405	1.6	–	–
Camphene	954	132,788,539	4.4	–	–	50,393,658	1.9
Verbenene	967	–	–	–	–	27,774,886	1.1
Sabinene	975	44,039,171	1.5	–	–	–	–
β-pinene	979	29,556,997	1.0	–	–	–	–
β-myrcene	988	29,379,228	1.0	–	–	–	–
Yomogi alcohol	999	41,103,264	1.4	–	–	32,349,759	1.2
*p*-cymene	1024	–	–	–	–	68,643,760	2.6
1,8-cineole	1026	368,453,758	12.3	–	–	142,491,224	5.4
Limonene	1029	–	–	1,520,194,907	4.3	–	–
*Cis*-ocimene	1037	–	–	3,045,372,978	8.7	–	–
*Trans*-ocimene	1050	–	–	2,654,890,107	7.6	–	–
ɣ-terpinene	1059	–	–	338,598,016	1.0	–	–
Artemisia ketone	1062	784,989,266	26.2	–	–	–	–
Artemisia alcohol	1083	33,487,881	1.1	–	–	–	–
α-thujone	1102	–	–	–	–	221,078,863	8.4
β-thujone	1114	–	–	–	–	418,253,095	15.9
Camphor	1146	576,552,736	19.2	–	–	663,407,985	28.3
Isoborneol	1160	–	–	–	–	46,469,992	1.8
*p*-mentha-1(7),2-dien-8-ol	1168	–	–	–	–	41,176,697	1.6
Borneol	1169	28,483,988	1.0	–	–	110,459,948	4.2
4-terpineol	1177	33,586,786	1.1	–	–	56,816,893	2.2
Estragole	1196	2.3695E + 10	67.6	23,695,362,067	67.6	–	–
Verbenone	1205	–	–	–	–	36,006,336	1.4
Cuminic aldehyde	1239	–	–	389,019,146	1.1	–	–
*Cis*-chrysanthenyl acetate	1256	–	–	–	–	33,128,295	1.3
Bornyl acetate	1285	–	–	–	–	41,891,342	1.6
Thymol	1289	–	–	–	–	43,935,135	1.7
Carvacrol	1298	–	–	–	–	28,233,145	1.1
α-copaene	1376	47,639,328	1.6	–	–	–	–
*Trans*-caryophyllene	1419	136,219,018	4.5	–	–	–	–
Germacrene D	1481	84,811,438	2.8	–	–	–	–
β-selinene	1490	90,133,377	3.0	–	–	–	–
Methoxycinnamaldehyde	1528	–	–	522,580,078	1.5	–	–
Caryophyllene oxide	1583	35,663,606	1.2	–	–	35,787,170	1.4

### Physical properties of the nanoliposomes

**Figure 1:**
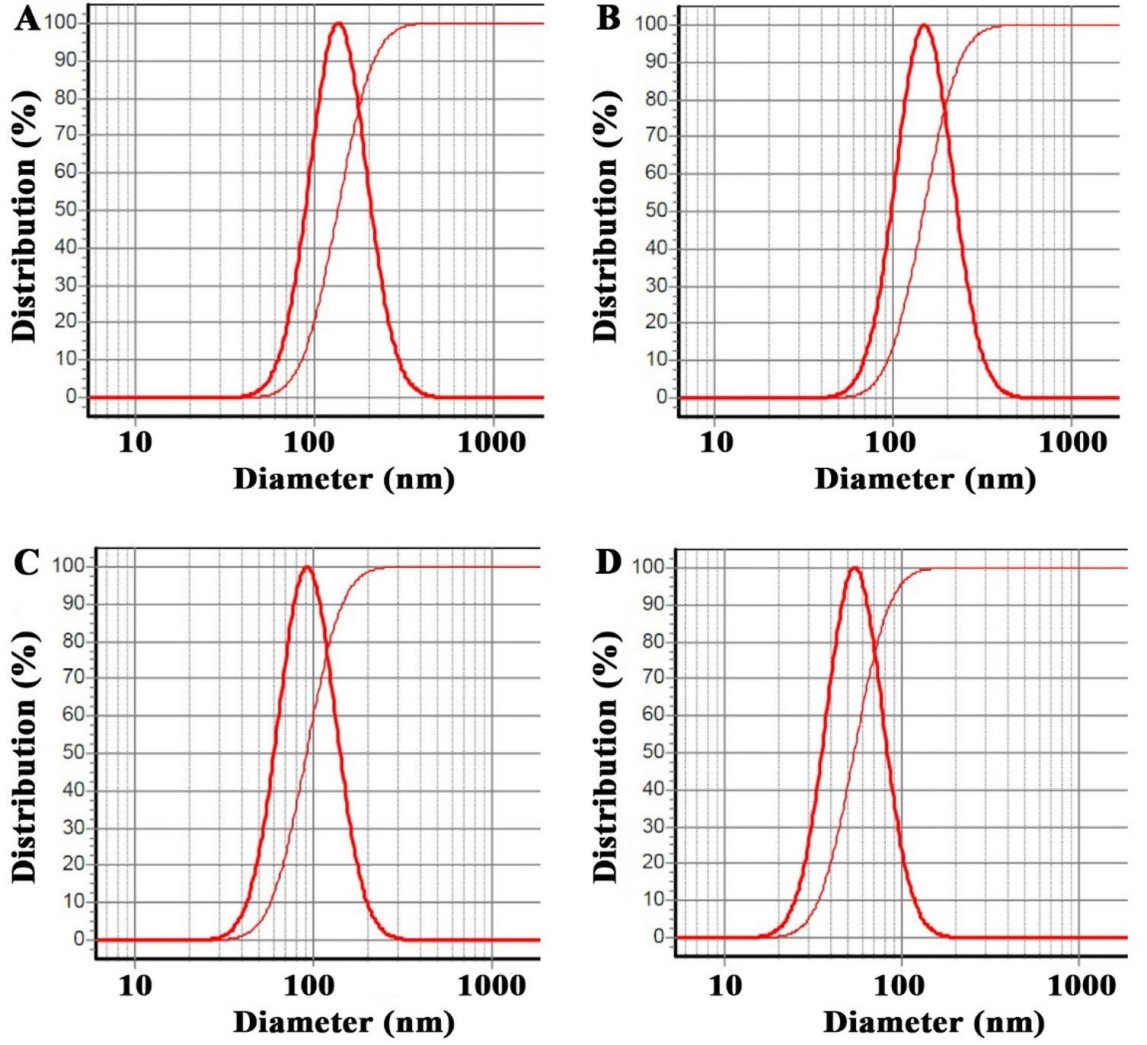
DLS diagrams of nanoliposomes containing EOs of *A. annua*, *A. dracunculus*, and *A. sieberi* and free nanoliposomes (**A**–**D**) with particle sizes of 137 ± 5, 151 ± 6, 92 ± 5, and 55 ± 4 nm.

**Figure 2:**
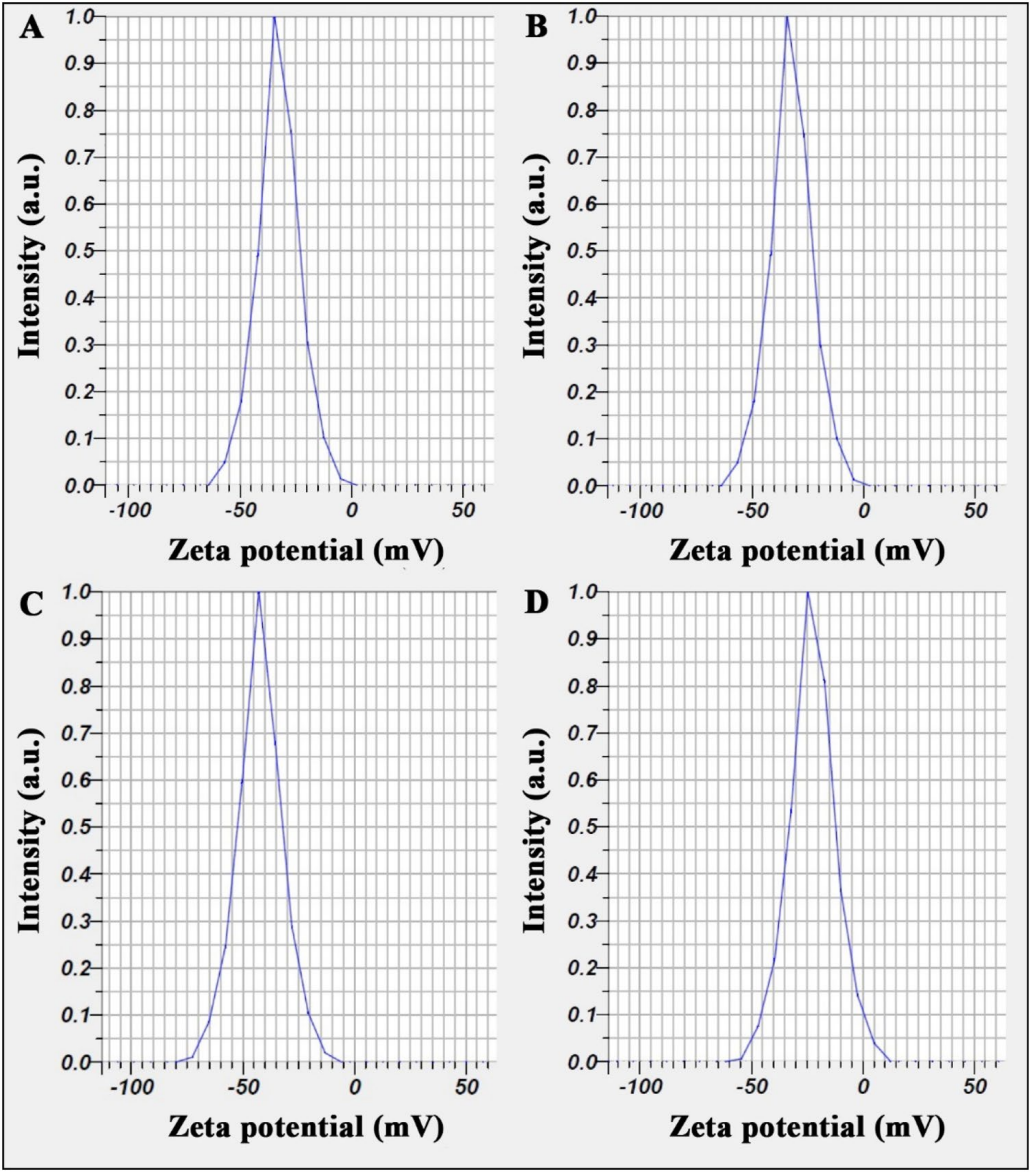
Zeta potential diagrams of nanoliposomes containing EOs of *A. annua*, *A. dracunculus*, and *A. sieberi* and free nanoliposomes (**A**–**D**) with particle sizes of 32 ± 0.5, 32 ± 0.6, 43 ± 1.7, and 23 ± 1.2 nm.

DLS diagrams of nanoliposomes containing EOs of *A. annua*, *A. dracunculus*, and *A. sieberi* and free nanoliposomes are depicted in Fig. 1A–D. Particle sizes were obtained as 137 ± 5, 151 ± 6, 92 ± 5, and 55 ± 4 nm, and their SPAN values were 0.96, 0.97, 0.97, and 0.96. Moreover, their potential zeta diagrams are shown in Fig. 2A–D; obtained values were 32 ± 0.5, 32 ± 0.6, 43 ± 1.7, and 23 ± 1.2 mV.

Furthermore, due to the high concentration, the sedimentation in all three nanoliposomes started after about 6 h. After overnight, two-phase suspensions were observed, with a clear supernatant and an agglomerate of nanoparticles below; no oily phases were observed at the top of the solutions. Agglomerate differs from aggregate; in the first one, the boundary between the nanoparticles is preserved and can be re-dispersed. In the next one, the nanoparticles become one and cannot be re-dispersed easily^36,37^. However, due to the presence of surfactant in the prepared nanoliposomes, these suspensions were re-dispersed with a simple shake, and their size did not change much from the initial state (data not shown). It is added that because the preparation site of nanoliposomes (Fasa University of Medical Sciences, Iran) and the larvicide testing site (Hormozgan University of Medical Sciences, Iran) are about 500 km away, the larvicide tests were conducted after 6 months of preparation. Therefore, it can be concluded that this re-dispersion did not affect nanoliposome efficacy. However, if the larvicidal test could have been performed immediately after preparation, the results would have been more accurate. However, in practical conditions, a larvicide usually is used for several months or years after manufacturing, so when the efficacy of these nanoliposomes was proper after six months, it can be concluded that they have good stability.

### Successful loading of the EOs in the nanoliposomes

**Figure 3:**
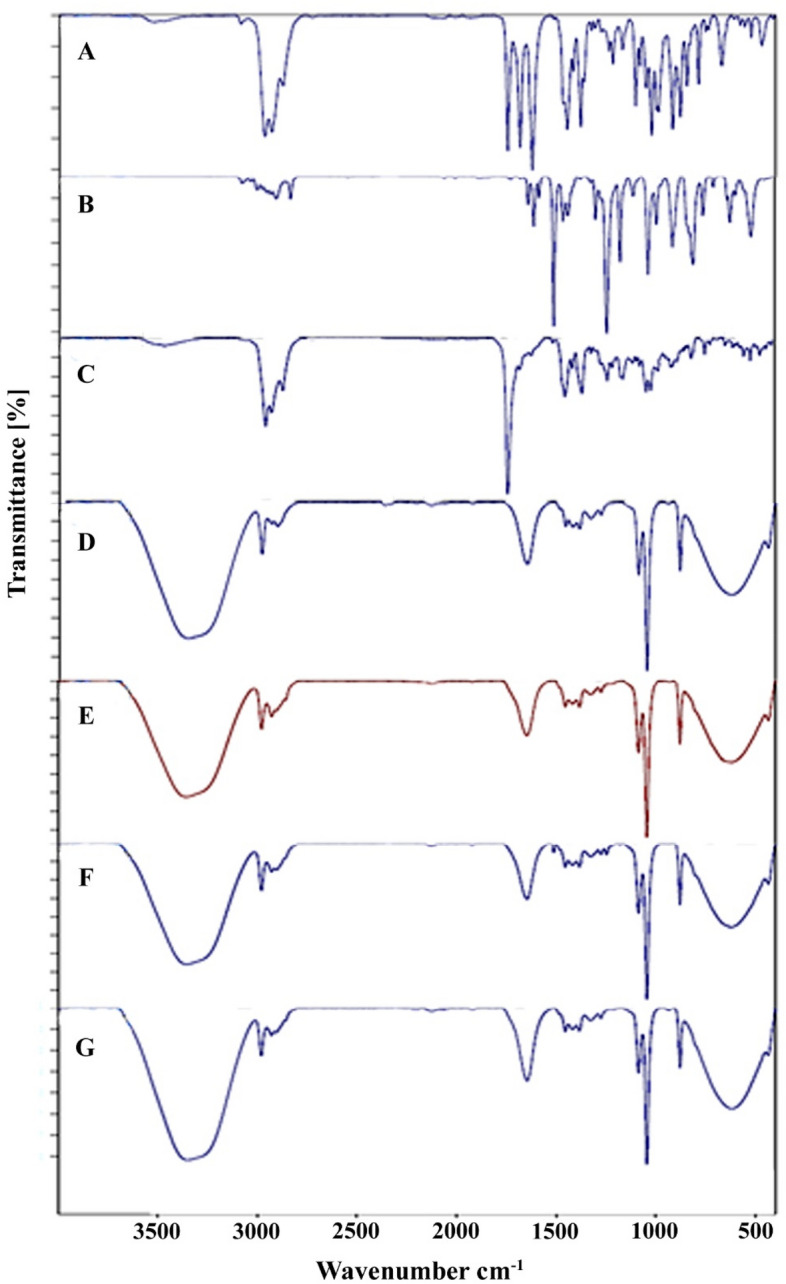
ATR-FTIR spectra of EOs of *A. annua*, *A. dracunculus*, and *A. sieberi* and free nanoliposomes (**A**–**D**), and nanoliposomes containing EOs of *A. annua*, *A. dracunculus*, and *A. sieberi* (**E**–**G**).

ATR-FTIR spectrum of *A. annua* EO (Fig. 3A) displayed the broadband at 3514 cm^−1^ attributed to OH and the characteristic peaks at 3084 and 3028 cm^−1^ can be related to C–H SP^2^ hybrid of alkyne. Besides, the spectra at 2963 and 2928 cm^−1^ corresponded to –CH stretching vibration due to alkanes, the spectra at 2872 and 2725 cm^−1^ indicating CH stretching vibration in aldehyde structure, the spectrum at 1743 cm^−1^, related to C=O, and the spectra at 1646, 1445 cm^−1^ can be related to C=C in aromatic compounds. The peak at 1445 cm^−1^ is attributed to the bending vibration of alcohol C–OH. The peaks at 1167, 1071, and 1004 cm^−1^ can be related to the stretching vibrations of C–O. The spectrum at 970 cm^−1^ is allocated to =C–H out-of-plane bending vibration from aromatics, and the characteristic band at 749 cm^−1^ is attributed to C–H vibration in benzene.

ATR-FTIR spectrum of *A. dracunculus* EO (Fig. 3B) displayed the peaks at 3076 and 3032 cm^−1^; they are attributed to SP^2^ hybrid of alkyne, the spectra at 2976, 2953, 2933, 2906, and 2834 cm^−1^ displayed –CH stretching vibration in SP^3^. The characteristic band at 1727 and 1638 cm^−1^ can be allocated to carbonyl groups. The band at 1509 cm^−1^ can be related to the C=C vibration in the aromatic ring and the characteristic band at 1243 cm^−1^ is attributed to C–O stretching vibration. Besides, the spectrum at 1035 cm^−1^ can be related to C–H bending absorption; also, the spectrum at 808 cm^−1^ is allocated to C–H vibration in benzene.

ATR-FTIR spectrum of *A. sieberi* EO (Fig. 3C) indicated the spectrum at 3467 cm^−1^ assigned to OH stretching due to phenolic compound in the EO. The characteristic peaks at 2958, 2924, and 2872 m^−1^ are ascribed to C–H stretching due to aliphatic compound, and the strong band at 1741 corresponded to (C=O), Carbonyl stretch representing aldehyde or ketones. The peak at 1454 cm^−1^ exhibited CH_2_ bending, and the absorption at about 1367 cm^−1^ is allocated to CH_3_ bending.

ATR-FTIR spectrum of free liposome (Fig. 3D) displayed the broad band between 3200 and 3600 cm^−1^ attributed to the presence of the hydroxyl group (OH), and the spectra at 2977, 2929, and 2900 cm^−1^ are attributed to C–C–H stretching. The absorption at 1645 cm^−1^ corresponded to the presence of the carbonyl group, and the spectrum at 1453 cm^−1^ indicates CH_2_ bending. Besides, the absorption at around 1383 cm^−1^ can be related to CH_3_ bending. The characteristic spectrum at 1085 cm^−1^ confirmed that the presence of P=O, the absorption at 1044 cm^−1^ could be related to C–O stretching, the characteristic absorption at 934 cm^−1^ corresponded to N(CH_3_)_3_, and the spectrum at 877 cm^−1^ represented the P–O stretching due to presence of lecithin.

It is evidenced from the blank and liposome containing EO for all absorption bands of interest that little difference was observed because the functional group of EO overlapped with the strong bands of the blank liposome.

ATR-FTIR spectrum of liposome containing *A. annua* EO (Fig. 3E) represented the broad and characteristic peak between 3200 and 3600 cm^−1^ attributed to the hydroxyl group due to hydrogen bonding between plant phenolic compound in the EO, carbonyl, and phosphate groups of lecithin. The absorptions at 2977 and 2929, 2900 cm^−1^ are allocated to symmetric and anti-symmetric vibration of CH_2_ in the alkyl chain in EO, tween 20, lecithin, and cholesterol. The spectrum at 1645 cm^−1^ is attributed to the carbonyl group. The phosphate stretching in 1274 and 1085 cm^−1^ is attributed to the interaction of compounds in the EO and fatty acid chains or polar heads in the nanoliposome.

ATR-FTIR spectrum of liposome containing *A. dracunculus* EO (Fig. 3F) displayed the broad band between 3200 to 3600 cm^−1^ allocated to the OH group due to hydrogen bonding between carboxyl and phosphate groups of lecithin. The absorption at 2976 and 2928 cm^−1^ are allocated to C-H starching due to SP^3^ hybrids of alkane in EO, tween 20, lecithin, and cholesterol. The spectrum at 1644 cm^−1^ can be attributed to the C=O. The phosphate stretching is presented in 1274 and 1085 cm^−1^, and the spectrum at 1044 cm^−1^ is attributed to C–O.

ATR-FTIR spectrum of liposome containing *A. sieberi* EO (Fig. 3G) showed the broadband between 3200 to 3600 cm^−1^ corresponded to hydroxyl groups due to hydrogen bonding interaction between EO, carbonyl, and phosphate groups. Besides, The spectra at 2978 and 2927 cm^−1^ allocated to C–H starching vibration related to alkanes in EO, Lecithin, tween 20, and cholesterol. Besides, the peak at 1644 cm^−1^ can be attributed to the C=O. The phosphate stretching presented in 1274 and 1085 cm^−1^ can be allocated to the interaction of compounds in EO and fatty acid chains or polar heads in nanoliposome.

### Larvicidal effects of the nanoliposomes

**Figure 4:**
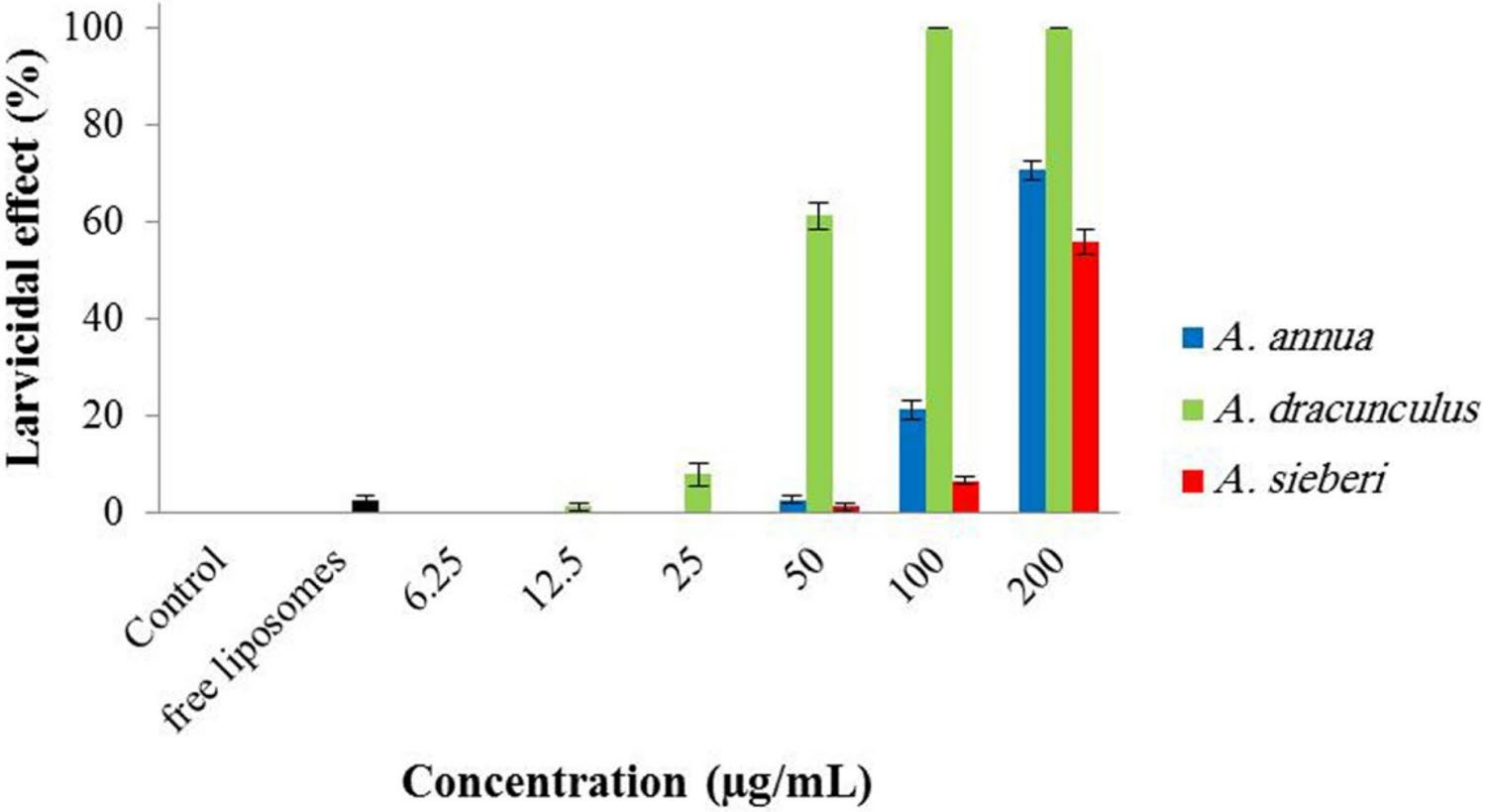
Larvicidal effects of free nanoliposomes and nanoliposomes containing EOs of *A. annua*, *A. dracunculus*, and *A. sieberi* against *Ae. aegypti.*

Larvicidal effects of free nanoliposomes and nanoliposomes containing EOs of *A. annua*, *A. dracunculus*, and *A. sieberi* against *Ae. Aegypti* is illustrated in Fig. 4. Free liposomes showed negligible effects on the viability of larvae (~ 2%). Interestingly, nanoliposomes containing *A. dracunculus* inferred complete larvicidal effects (100%) at 100 and 200 µg/mL. Additionally, nanoliposomes containing *A. annua* and *A. sieberi* EOs caused 70 and 56% larval mortality at 200 µg/mL.

**Figure 5:**
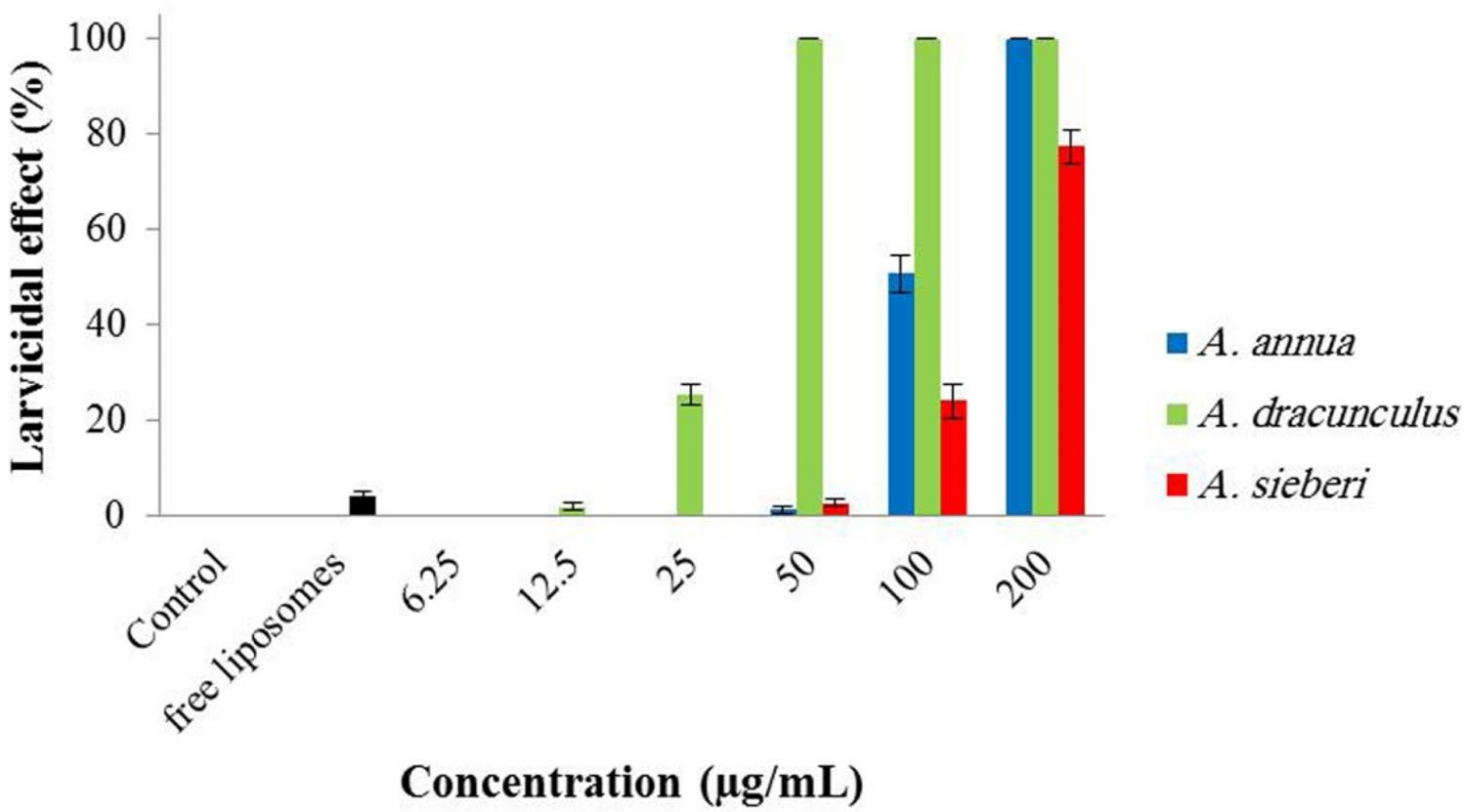
Larvicidal effects of free nanoliposomes and nanoliposomes containing EOs of *A. annua*, *A. dracunculus*, and *A. sieberi* against *An. stephensi.*

The larvicidal effects of the samples against *An. stephensi* is shown in Fig. 5. Free liposomes showed negligible effects on the viability of larvae (~ 4%). Besides, nanoliposomes containing *A. sieberi* EOs caused 77% larval mortality at 200 µg/mL. Interestingly, perfect larvicidal effects were observed after treatment with nanoliposomes containing *A. dracunculus* EO (50, 100, and 200 µg/mL) and nanoliposomes containing *A. annua* EO (200 µg/mL).

Obtained LC_50_ values of samples against *Ae. aegypti* and *An. stephensi* are summarized in Table 2. Nanoliposomes containing *A. dracunculus* EO with LC_50_ value of 34 (25–46) µg/mL against *Ae. aegypti* was significantly more potent (*P* < 0.05) than nanoliposomes containing *A. annua* and *A. sieberi* with LC_50_ values of 151 (149–153) and 197 (152–255) µg/mL. Moreover, the efficacy of nanoliposomes containing *A. dracunculus* EO against *An. stephensi* was significantly more potent (*P* < 0.05) than the others. LC_50_ values in order of efficacy were 23 (16–31), 90 (77–103), and 140 (138–141) µg/mL; these values were related to nanoliposomes containing *A. dracunculus, A. annua,* and *A. sieberi*.

**Table 2:** LC_50_ values (µg/mL) of nanoliposomes containing EOs of *A. annua*, *A. dracunculus*, and *A. sieberi* against examined larvae.

Species	*A. annua*	*A. dracunculus*	*A. sieberi*
*Ae. aegypti*	151	34	197
	(149–153)*	(25–46)	(152–255)
*An. stephensi*	90	23	140
	(77–103)	(16–31)	(138–141)

*Lower and Upper confidence limits.

## Discussions

Excessive use of synthetic pesticides has led to environmental pollution with enhancement in vectors’ resistance^38,39^. The development of resistance to insecticides such as pyrethroids, organophosphates, organochlorines, and carbamates has precluded the successful elimination of larval stages^40,41^. For instance, a high rate (78%) of pyrethroids resistance in the WHO African Region has been demonstrated^42^. EOs consist of various natural volatile hydrocarbons and phenylpropenes molecules^43^. Monoterpenes are the main component of EOs which exert neurotoxic effects on insects via AChE and GABA activities^16,44^. However, total EOs confers substantially higher larvicidal or insecticidal effects through multi-target effects^45,46^. So in this study, three EOs from *Asteraceae* Family was used as larvicides. *A. annua* L. is a polyphenols-reach plant with antimalarial effects that grows in various geographical and soil pH conditions^47^. Another member of the *Asteraceae* Family, *A.dracunculus* has demonstrated larvicidal, antimicrobial, anticancer, and anti-inflammatory effects^48,49^. In addition, *Artemisia sieberi* has exhibited antimicrobial, antifungal, larvicidal, and insecticidal traits^50–52^.

In recent years many reports on using nanostructures containing EOs as mosquito repellents or larvicides have been published. For instance, nanogel containing *Zataria multiflora* EO with 600 min repellent against *An. stephensi* compared to 242 min efficacy of DEET^53^. Moreover, nanoemulsion of *Cinnamomum zeylanicum* EO (55 ppm) caused 92% larval mortality against *An. stephensi*^54^. Besides, The perfect larvicidal effect against *Cx. quinquefasciatus* was achieved at 4 h of exposure with nanoemulsion *Eucalyptus globulus* EO^55^. Nowadays, it is accepted that the efficacy of nanoformulation is more than the non-formulated state of EO. For instance, the LC_50_ value of *Mentha piperita* EO against *Cx. pipiens* in its nanoemulsion state compare to the non-formulated state decreased from 88.90 to 31.24 μg/mL^56^. By the way, the LC_50_ value of *Siparuna guianensis* EO against *Ae. aegypti* decreased from 86.52 to 24.75 μg/mL in its nanoemulsion state^57^. The smaller the nanoparticle size, the greater the mobility, and more collisions with larvae (due to Brownian motion) led to better accessibility, permeability, and toxicity against the larval body^58,59^. The current study demonstrated nanoliposomes containing *A. dracunculus* with LC_50_ values of 34 and 23 μg/mL against *Ae. aegypti* and *An. stephensi* is a great formulation for use as a mosquito larvicide. Its efficacy is also more than many available reports. For instance, nanoemulsion containing *Pterodon emarginatus* at 250 μg/mL showed 100% larvicidal effects on *Ae. aegypti*^60^. Besides, the LC_50_ value of *Lippia alba* nanoemulsion against *Ae. aegypti* was 31.02 μg/mL^61^. Moreover, the LC_50_ value of *Myrtus communis* nanoemulsion against *An. stephensi* was reported as 26.1 μg/mL^62^. Besides, *Eucalyptus globulus* EO nanogel against *An. stephensi* was reported as 32 μg/mL^63^. The main reason for its considerable capability might be related to the high percentage of estragole (67.6%), which can be used as a larvicidal agent for mosquito control programs. In that way, our findings provide a possible way for further studies to find out the active molecule. However, further investigations must be conducted to describe the mode of action of each constituent independently.

## Conclusions

The chemical compositions of three used EOs were first investigated. Artemisia ketone (26.2%), camphor (19.2%), 1,8-cineole (12.3%), trans-caryophyllene (4.5%), and camphene (4.4%) included five major compounds of *A. annua* EO. Besides, estragole (67.6%), *cis*-ocimene (8.7%), ɣ-terpinene (7.6%), *trans*-ocimene (4.3%), and α-pinene (1.6%) were five major compounds in *A. dracunculus* EO. Moreover, camphor (28.3%), β-thujone (15.9%), α-thujone (8.4%), 1,8-cineole (1.8%), and borneol (4.2%) were five major compounds in *A. sieberi* EO. Larvicidal effects of nanoliposomes containing each EO against *An. stephensi* or *Ae. aegypti* were then investigated. Considering the promising results of nanoliposomes containing *A. dracunculus* EO against two important medical species, it could be a distinct candidate against other mosquitos’ larvae.

## Data Availability

The data used to support the findings of this study are included within the article.
